# An Internet-Based Guided Self-Help Intervention for Panic Symptoms: Randomized Controlled Trial

**DOI:** 10.2196/jmir.2362

**Published:** 2013-07-29

**Authors:** Wouter van Ballegooijen, Heleen Riper, Britt Klein, David Daniel Ebert, Jeannet Kramer, Peter Meulenbeek, Pim Cuijpers

**Affiliations:** ^1^Department of Clinical Psychology and the EMGO Institute for Health and Care ResearchVU University AmsterdamAmsterdamNetherlands; ^2^Innovation IncubatorLeuphana UniversityLüneburgGermany; ^3^DVC-Research Portfolio & the Faculty of Health SciencesUniversity of BallaratBallaratAustralia; ^4^Centre of Mental Health ResearchThe Australian National UniversityCanberraAustralia; ^5^National eTherapy CentreFaculty of Life and Social SciencesSwinburne UniversityMelbourneAustralia; ^6^Philipps University MarburgMarburgGermany; ^7^Innovation Centre of Mental Health & TechnologyTrimbos-instituteUtrechtNetherlands; ^8^GGNet (Community Mental Health Centre)WarnsveldNetherlands; ^9^Department of Psychology, Health and TechnologyUniversity of TwenteEnschedeNetherlands

**Keywords:** Internet, self-help, panic disorder, anxiety disorders, patient adherence

## Abstract

**Background:**

Internet-based guided self-help is efficacious for panic disorder, but it is not known whether such treatment is effective for milder panic symptoms as well.

**Objective:**

To evaluate the effectiveness of Don’t Panic Online, an Internet-based self-help course for mild panic symptoms, which is based on cognitive behavioral principles and includes guidance by email.

**Methods:**

A pragmatic randomized controlled trial was conducted. Participants (N=126) were recruited from the general population and randomized to either the intervention group or to a waiting-list control group. Inclusion criteria were a Panic Disorder Severity Scale-Self Report (PDSS-SR) score between 5-15 and no suicide risk. Panic symptom severity was the primary outcome measure; secondary outcome measures were anxiety and depressive symptom severity. Measurements were conducted online and took place at baseline and 12 weeks after baseline (T1). At baseline, diagnoses were obtained by telephone interviews.

**Results:**

Analyses of covariance (intention-to-treat) showed no significant differences in panic symptom reduction between groups. Completers-only analyses revealed a moderate effect size in favor of the intervention group (Cohen’s d=0.73, *P*=.01). Only 27% of the intervention group finished lesson 4 or more (out of 6). Nonresponse at T1 was high for the total sample (42.1%). Diagnostic interviews showed that many participants suffered from comorbid depression and anxiety disorders.

**Conclusions:**

The Internet-based guided self-help course appears to be ineffective for individuals with panic symptoms. However, intervention completers did derive clinical benefits from the intervention.

**Trial Registration:**

Nederlands Trial Register: NTR1639; http://www.trialregister.nl/trialreg/admin/rctview.asp?TC=1639 (Archived by WebCite at http://www.webcitation.org/6ITZPozs9).

## Introduction

Panic disorder (PD) with or without agoraphobia is a prevalent anxiety disorder associated with substantial loss of quality of life for the patient and considerable costs to society [1-4]. Subclinical PD, defined as panic symptoms that do not meet full *Diagnostic and Statistical Manual of Mental Disorders* (Fourth Edition; *DSM-IV*) criteria for PD, is just as prevalent [2,4]. Subclinical panic symptoms can develop into clinical PD and are also a predictor for the development of mental disorders other than PD, such as generalized anxiety disorder, social phobia, or major depressive disorder (MDD) [5].

For treatment, PD can be effectively treated with psychological or drug therapy [6-8]. Research indicates that it is also possible to prevent or delay the onset of clinical PD in people with subclinical panic symptoms [9,10]. A recent study showed that a group intervention involving primarily cognitive behavioral therapy effectively reduced symptoms in subclinical cases of PD, as well as in relatively mild cases [10]. This group course, called Don’t Panic, could also be acceptable from a cost-effectiveness point of view [11].

Internet-based guided self-help has shown to be an efficacious treatment of PD as well, with a large effect size (Hedge’s *g*=0.83) [12]. To date, all but 1 study [13] comparing Internet-based guided self-help for PD with a control condition have focused purely on groups with clinical PD, which commonly was also the primary diagnosis (eg, [14,15]). These studies excluded subclinical cases (eg, [14-16]). Recently, an Internet-based version of the group course Don’t Panic has been developed. This intervention, Don’t Panic Online, is an Internet-based self-help course with minimal guidance specifically for individuals with mild panic symptom severity. The aim was to provide an accessible, low-intensity, early intervention for panic symptoms.

The current study is a pragmatic randomized controlled trial (RCT) of the effectiveness of Don’t Panic Online in reducing panic and anxiety symptoms among participants with subclinical and mild clinical PD. We postulate a difference in effect between Don’t Panic Online and a waiting-list control group.

## Methods

### Design

We conducted a pragmatic RCT with 2 arms: (1) Internet-based guided self-help, and (2) a waiting-list control group (see subsequent description). The Medical and Ethical Committee of VU University Medical Center approved the study protocol, which is described in greater detail elsewhere [17]. This paper was written in accordance with the CONSORT-EHEALTH checklist [18], and this trial has been registered in the Netherlands Trial Register (NTR1639). The Netherlands Trial Register is part of the Dutch Cochrane Centre.

### Study Population

We included participants aged 18 and older, with subclinical PD or clinical PD with relatively mild symptom severity, who had access to the Internet. Any individuals who were at risk of suicide were excluded. Subclinical or mild PD was defined as having a score ranging from 5 to 15 on the Panic Disorder Severity Scale-Self Report (PDSS-SR) [19]. These cut-off points represent slight to moderate panic symptom severity [20]. No restrictions were imposed on the use of pharmacotherapy or psychotherapy.

### Sample Size

Previous RCTs of Internet-based self-help interventions for panic symptoms showed large between-group effect sizes [12]. Our aim was to recruit participants with milder symptom severity than those who took part in these studies. Therefore, our sample was expected to show a smaller decrease in panic symptoms. Based on a moderate effect size (Cohen’s *d* [*d*]= 0.50), and using a 2-sided *t* test (alpha = .05, power 80%) to compare the PDSS-SR scores of the intervention group with those of the control group, we aimed to include 128 participants [21], with 64 in each group. Any missing values at posttreatment were imputed.

### Recruitment

Participants were recruited from the general population. Most of those who applied for participation did so after reading about this study in the health section of an online newspaper. Additional online recruitment was conducted by means of a Facebook advertising campaign and by posting messages on panic-related or anxiety-related message boards. This was supplemented by offline recruitment by means of advertisements in national newspapers and articles in local newspapers. Interested individuals were directed to a study website, where they could find information about participation and a downloadable informed consent form. The application procedure involved printing and signing the informed consent form, then sending this to the research team (either as a physical document, by conventional mail, or as a scanned document attached to an email).

### Randomization and Procedure

Consenting applicants were sent an email with a link to the online questionnaires. The baseline (T0) questionnaires included the screening questionnaires for inclusion. Any participants who reported severe panic symptoms or who were at risk of suicide were sent an automatic message advising them to contact their general practitioner and/or to visit a website for suicide prevention. This website [22] offers psychoeducation and a helpline by telephone or online chat [23]. Those participants who had completed T0 and who met the inclusion criteria were contacted within 2 weeks for a diagnostic interview by telephone. This interview was used to obtain a more detailed overview of the study sample, not for the purposes of inclusion or exclusion. After the interview, all participants were randomized to 1 of the 2 groups. Randomization was stratified for the presence or absence of agoraphobic symptoms (PDSS-SR item 4 score ≥2) and the use of antidepressants or sedatives. Randomization lists were generated automatically using a computer program. The T0 measurement can be considered to be double blind because the participants were not randomized until they had completed all of the questionnaires and the diagnostic interview. Blinding of the participants at posttreatment assessment (T1) was not possible because at that stage they were aware of the nature of the group to which they had been allocated. T1 was scheduled 12 weeks after the baseline assessment. Both T0 and T1 were self-reported and were conducted through the Internet. Any participants who had not completed T0 or T1 were sent up to 3 automated reminders by email at weekly intervals.

### Intervention

Don’t Panic Online is a guided, Internet-based, individual, self-help course, based on cognitive behavioral therapy principles. The course consists of 6 sessions in which the participants learn to control their panic symptoms by applying various cognitive and behavioral techniques and skills. The course’s content is described in more detail elsewhere [17]. A typical lesson takes approximately 30 minutes and consists of an introduction, a discussion of the previous lesson’s homework, new theory, and homework for the following week. A track-and-trace system keeps a record of the dates on which participants log on and complete a lesson. The participants in the intervention group were coached by trained, Master’s-level clinical psychology students. Every week, these participants received an email from their coach, asking how they were doing and whether they were experiencing any difficulty in following the program. The coaches responded to questions about the course and the associated exercises. They also gave brief replies to questions about the participant’s mental health. The coaches were supervised by the first author. On average, the total time spent on each participant was 1 to 2 hours.

Participants in the control group received access to Don’t Panic Online after completing the T1 measurement (12 weeks after T0). While waiting, they had access to an information website about the symptoms of panic and agoraphobia. This website included advice to contact a general practitioner in case the participant had further questions about panic symptoms and its treatment. All participants in the control group and the intervention group were free to seek any (additional) help they might require.

### Instruments

The following variables were measured: demographic data, *DSM-IV* diagnosis, symptoms of anxiety and panic, depressive symptoms, and suicide risk. All variables were measured at both T0 and T1, except for demographic data, diagnosis, and suicide risk, which were only measured at T0.

The T0 measurement started with demographic questions. These included age, gender, place of birth, marital status, education level, physical health, and previous mental health diagnoses.

The Composite International Diagnostic Interview (CIDI) 12-month prevalence [24] was used to ascertain the presence or absence of PD, other anxiety disorders, and depression. A clinical diagnosis was made, not as an inclusion criterion, but to gain a more complete overview of the participants. The CIDI, which was developed by the World Health Organization, is an extensive, fully structured, diagnostic interview to assess *DSM-IV* Axis-I diagnoses [24]. The only subscales used were depression, PD, agoraphobia, generalized anxiety disorder, social phobia, and posttraumatic stress disorder. In this study, a trained interviewer administered the CIDI by telephone.

The severity of current panic symptoms was measured using the PDSS-SR. The PDSS, which was originally designed as a face-to-face interview for both research and clinical practice [25], was adapted to be used in a patient self-report format [19]. The instrument contains 7 items that assess the severity of 7 dimensions of PD and its associated symptoms. The PDSS-SR generates a total score ranging from 0 to 28. The higher the score, the more severe the panic symptoms. The questionnaire has adequate psychometric properties when compared with the PDSS [19,26]. For the purposes of the current study, a score of less than 5 indicates that there are no clinically significant symptoms, whereas a score of more than 15 is interpreted as severe PD. Therefore, our study focused on the group with scores ranging from 5 to 15. According to the study by Furukawa et al [20], this score range identifies participants with mild to moderate panic symptoms but excludes those without panic symptoms as well as those with severe panic symptoms.

Anxiety symptoms in general were measured using the Beck Anxiety Inventory (BAI) [27]. The BAI contains 21 short questions. Convergent and divergent validity is sufficient [28,29]. The score ranges from 0 to 63. A score of 30 or more is considered to correspond to severe anxiety symptoms.

Depressive symptoms were measured using the Center for Epidemiologic Studies Depression scale (CES-D) [30]. The CES-D is a 20-item self-report questionnaire. The score of each individual item ranges from 0 to 3, whereas the total score ranges from 0 (no feelings of depression) to 60 (severe feelings of depression). Convergent validity of the online Dutch version for adults with other depressive measures is good [31]. With a cut-off score of 22 for MDD, it also has good predictive validity [31].

Suicide risk and suicidal ideation were measured using the specific section of the Mini-International Neuropsychiatric Interview (MINI) [32,33]. The MINI suicide section consists of 6 items and classifies participants into categories ranging from no suicide risk to high suicide risk. Any individuals with a moderate to high suicide risk were excluded from this study. In the current study, these items were administered online and presented as self-report items.

An indication of health care services usage during the past month was obtained using Part I of the Trimbos and Institute of Medical Technology Assessment Questionnaire on Costs Associated with Psychiatric Illness (TiC-P) [34].

Finally, the T1 battery of online questionnaires included open questions concerning the participant’s subjective experience with Don’t Panic Online and reasons for not finishing the program. These questions were only administered to the intervention group.

### Analyses

Firstly, means and standard deviations were calculated for age and symptom severity of panic, anxiety, and depression. Any differences in symptom severity between the intervention group and control group were expressed in terms of Cohen’s *d* (see subsequent description) to give an indication of the magnitude of the difference in question.

Between-group effects at T1 were calculated using analyses of covariance (ANCOVA), controlling for pretreatment scores. Instead of *F* values, *t* values of parameter estimates are reported because only 2 groups are compared (where *t* ^2^ = *F*). Effect sizes on continuous measures were expressed in terms of Cohen’s *d*, which was calculated by and dividing the mean difference between the 2 mean scores by the pooled standard deviation: (mean_1_–mean_0_)/SD_Pooled_. Effect sizes of 0.8 can be assumed to be large, whereas effect sizes of 0.5 are moderate and effect sizes of 0.2 are small [21]. Because Cohen’s *d* does not take covariance into account, partial *η* ^2^ is also reported in this paper. It cannot be estimated which level of partial *η* ^*2*^ could be considered adequate because this effect size is dependent on several factors. Within-group effects were analyzed using paired-sample *t* tests and expressed in terms of Cohen’s *d* in which the correlation between T0 and T1 was taken into account by applying Morris and DeShon’s equation 8 [35]. Finally, the proportion of participants below the PDSS-SR cut-off points for clinical and subclinical PD was calculated for both T0 and T1. We used the cut-off points of 8 and 5, indicating clinical PD [25] and subclinical PD [20], respectively. All analyses were conducted for the full sample, for the subgroup completers, and for subgroups with and without the diagnosis of PD according to the CIDI. We maintained a 2-sided alpha of .05. For all analyses, SPSS version 17 (SPSS Inc, Chicago, IL, USA) was used.

The data were analyzed in agreement with the intention-to-treat (ITT) principle. Missing data at T1 were imputed by multiple imputation, in which all variables except for nominal variables (ie, age, education level, clinical diagnoses, and symptom severity on all measures at T0 and T1) were included as predictors. Ten datasets were generated and analyses were performed using pooled data. Compared with single imputation methods, multiple imputation generates a more conservative estimate of the sample standard error [36] and overestimation of effect sizes and *P* values is unlikely. For the purpose of sensitivity analysis, *P* values and effect sizes were also estimated by running the Expectation Maximization (EM) algorithm [37] on the missing data.

## Results

### Sample

**Figure 1:**
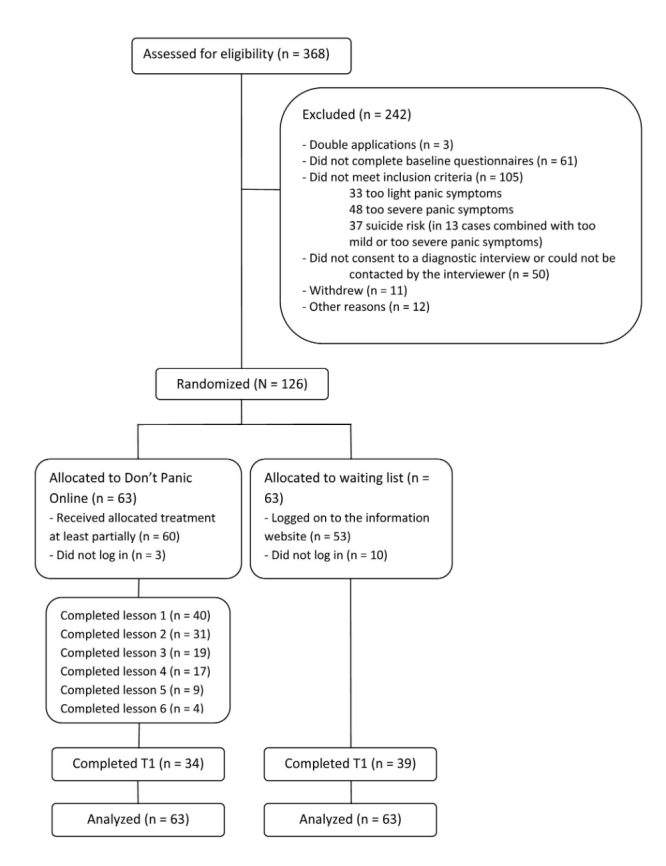
Flow of participants through the study.

Of 368 applicants who applied and sent in informed consent forms, 126 were included in the study. See Figure 1 for a flowchart and an overview of excluded applicants. The participants were primarily female (85/126, 67.5%), born in the Netherlands (115/126, 91.3%), with a mean age of 36.6 years (SD 11.4, range 18-67), and 50% had a bachelor’s degree or higher (Table 1). Diagnostic interviews showed that 97 (77.0%) of the included participants met the criteria for PD with or without agoraphobia. Other *DSM-IV* anxiety disorders and MDD were also prevalent (Table 1). Five participants (4.0%) did not meet the criteria for a diagnosis of a mood or anxiety disorder. The control group had slightly higher baseline scores than the intervention group (Table 1), but there were no to little further differences between the intervention group and control group. Details of health care services usage (eg, visits to the general practitioner) are presented in Table 2. Approximately half of the participants reported having consulted a general practitioner in the month immediately prior to the study, and one-third had seen a psychologist or psychiatrist.

### Study Dropout

The posttreatment measurement was completed by 73 participants (57.9%). There was no significant difference between the measurements and characteristics of these 73 study completers and those of the 53 participants who were lost to follow-up. However, within the intervention group, study dropouts were less likely to have completed lessons 1 to 4 of the course (χ^2^_1_ = 15.1, *P*<.001).

### Intervention Adherence

Of the 63 participants in the intervention group, 60 (95%) started lesson 1, whereas 3 participants did not log in at all (Figure 1). Approximately half of the participants (31/63, 49%) completed lesson 2. Five participants (8%) finished all 6 modules of Don’t Panic Online, 4 (6%) of them within the given 3-month time frame. During the trial, 3 participants (5%) reported that they experienced difficulties accessing the website. Those participants in the intervention group who completed T1 but did not complete the intervention (n=30) were asked why they dropped out. The most frequently reported reasons involved time constraints (n=13), life events (n=5), and symptoms so severe that the individual was unable to follow the program (or parts thereof) or carry out the assignments (n=5; see Table 3).

### Intention-to-Treat Analyses

After multiple imputation, ANCOVAs showed no significant difference in panic symptom severity at T1 between groups as measured by the PDSS-SR (*t* = –1.17, *P*=.25, partial *η* ^*2*^ = .023, *d*=0.30; Table 4). The within-group difference of the intervention group was significant (*t*=3.06, *P*=.007, *d*=0.62), as was the within-group difference of the control group, albeit with a smaller effect size (*t*=2.26, *P*=.03, *d*=0.40). The mean BAI score did not differ between groups (*t* = –1.71, *P*=.09, partial *η* ^*2*^ = .027, *d*=0.39; Table 4). Nor were there any differences between groups in terms of depressive symptoms, as measured by the CES-D (*t* = –1.56, *P*=.12, partial *η* ^*2*^ = .034, *d*=0.39; Table 4).

At T1, and with missing values imputed, 24 participants (38%) in the intervention group and 13 (20%) in the control group had PDSS-SR scores of less than 5 (ie, symptom free). This difference did not reach significance (χ^2^ = 5.7, *P*=.07). With regard to the cut-off point of 8 (the recommended cut-off for clinical diagnosis), 28 participants (44%) in the intervention group and 22 (35%) in the control group scored below 8 at T0. At T1, 38 participants in the intervention group (60%) and 33 participants in the control group (52%) scored below 8, a nonsignificant difference (χ^2^ = 1.3, *P*=.43).

Sensitivity analyses with the EM algorithm gave slightly different results. There was no significant effect between groups on the primary outcome measure (PDSS-SR: *t*_124_ = –1.79, *P*=.08, partial *η* ^*2*^ = .025, *d*=0.34), but the difference in BAI anxiety symptoms did reach significance (*t*_124_ = –2.33, *P*=.02) with a moderate effect size (*d*=0.46, partial *η* ^*2*^ = .042). CES-D depressive symptoms also differed between groups (*t*_124_ = –2.69, *P*=.008) with a moderate effect size (*d*=0.47, partial *η* ^*2*^ = .055).

**Table 1:** Baseline characteristics of participants.

Characteristics		Total sample N=126	Intervention group n=63	Control group n=63	Difference at baseline (Cohen’s *d*)
**Demographics**					
	Age, mean (SD)	36.6 (11.4)	36.7 (12.2)	36.4 (10.7)	
	Female, n (%)	85 (67.5)	44 (69.8)	41 (65.1)	
	Born in the Netherlands, n (%)	115 (91.3)	57 (90.5)	58 (92.1)	
	Living alone, n (%)	50 (39.7)	23 (36.5)	27 (42.9)	
	High education,^a^ n (%)	63 (50.0)	30 (47.6)	33 (52.4)	
	Physical health problems, n (%)	9 (7.1)	5 (7.9)	4 (6.3)	
	Previously diagnosed with a mental disorder, n (%)	47 (37.3)	22 (34.9)	25 (39.7)	
**Diagnoses,** ^b^ **n (%)** ^c^					
	PD with agoraphobia	61 (49.2)	30 (47.6)	31 (49.2)	
	PD without agoraphobia	36 (29.0)	17 (27.0)	19 (30.2)	
	Agoraphobia without PD	17 (13.7)	10 (15.9)	7 (11.1)	
	GAD	11 (8.9)	5 (7.9)	6 (9.5)	
	Social phobia	78 (62.9)	39 (61.9)	39 (61.9)	
	PTSD	16 (12.9)	4 (6.3)	12 (19.0)	
	MDD	53 (42.7)	27 (42.9)	26 (41.3)	
**Symptom severity, mean (SD)**					
	Panic (PDSS-SR)	8.9 (3.0)	8.8 (3.2)	9.1 (2.8)	0.12
	Anxiety (BAI)	24.9 (10.8)	23.7 (10.2)	26.0 (11.3)	0.22
	Depression (CES-D)	20.8 (9.0)	20.0 (9.1)	21.6 (9.0)	0.18

^a^Defined as the equivalent of a bachelor’s degree or higher.

^b^Percentages add up to more than 100% due to comorbid diagnoses.

^c^Missing data of 2 participants (n=124).

**Table 2:** Use of care in the past month.

Care use	T0, n (%)		T1,^a^ n (%)	
	Intervention group (n=63)	Control group (n=63)	Intervention group (n=16)	Control group (n=39)
Visited general practitioner	27 (43%)	31 (49%)	2 (13%)	12 (31%)
Visited psychologist or psychiatrist	23 (37%)	17 (27%)	5 (31%)	14 (36%)
Visited other professional health care giver	18 (29%)	25 (40%)	3 (19%)	14 (36%)
Used antidepressants, sedatives, or sleeping pills	20 (32%)	23 (37%)	7 (44%)	13 (33%)

^a^Differences within groups and between groups did not reach significance.

**Table 3:** Reasons why participants did not finish Don’t Panic Online within 12 weeks (n=30).

Reason for discontinuation	n^a^
Time constraint (too busy or need more time)	13
Life events (pregnancy, loss, family issues)	5
Symptoms too severe to do assignments	5
Found other therapy	4
Content not applicable	3
Spontaneous recovery	2
Adverse effect	1
More guidance needed	1
Lack of structure	1
Lessons too slow	1
Not motivated	1

^a^Numbers do not add up to 30 because 2 participants did not give reasons and others gave several.

**Table 4:** Differences between groups at T1, intention-to-treat (N=126).

Measure	Group, mean (SD)^a^		Between-groups effect^a^			
	Intervention group (n=63)	Control group (n=63)	Cohen’s *d* (95% CI)	ANCOVA^b^		
				*t* ^c^	*P*	Partial *η* ^*2*^
PDSS-SR	5.8 (4.9)	7.3 (4.9)	0.30 (–0.91, 1.51)	–1.17	.25	.023
BAI	17.0 (12.7)	22.0 (12.7)	0.39 (–2.74, 3.53)	–1.71	.09	.027
CES-D	16.4 (12.3)	21.1 (12.1)	0.39 (–2.59, 3.42)	–1.56	.12	.034

^a^Missing data imputed by multiple imputation.

^b^Controlling for symptom severity at T0.

^c^Degrees of freedom not provided due to multiple imputation.

### Completers-Only Analyses

Those participants in the intervention group who had completed the first 4 lessons (or more) of the course (n=17) were included in the completers-only analyses. These completers cannot be all considered to have completed the intervention, but after 4 lessons, participants can be considered to have experienced most of the content of the intervention. Sixteen of the 17 participants in the intervention group who had completed the first 4 lessons also filled in T1 questionnaires. Accordingly, there were 16 completers in the intervention group. These 16 individuals did not significantly differ from the noncompleters in the intervention group at T0 in terms of age, education, clinical diagnosis, and symptom severity. Control group completers were those who filled in T1 (n=39).

The ANCOVA showed significant differences between the intervention group completers and control group completers with regard to panic symptom severity at T1 (*t*_53_ = –2.60, *P*=.01, *d*=0.73; see Table 5), in favor of the intervention group. The intervention group was also characterized by a large within-group effect on panic symptoms (*t*_15_ = 4.92, *P*<.001, *d*=1.23). In the control group, within-group effects did not reach significance. ANCOVA also showed that BAI anxiety symptom severity differed significantly between groups (*t*_53_ = –2.37, *P*=.02, *d*=0.60, see Table 5), as did depressive symptom severity, as measured using the CES-D (*t*_53_ = –2.52, *P*=.02, *d*=.94).

Ten (68%) of the intervention completers and 8 (21%) of the control group completers had a PDSS-SR score of less than 5 at T1, which is a significant difference (χ^2^_1_ = 9.1, *P*=.003). In terms of the cut-off point for clinical diagnosis, 13 participants in the intervention group (81%) and 23 (59%) in the control group scored less than 8, but this difference did not reach statistical significance (χ^2^_1_ = 2.5, *P*=.12).

Lastly, health care service usage rates did not differ either within or between groups (see Table 2).

### Participants With Diagnosis of Panic Disorder Versus Those Without Diagnosis

Neither ITT nor completers-only analyses showed differences on any outcome measure between participants with and without clinical PD.

**Table 5:** Differences between groups at T1, completers^a^ (n=55).

Measure	Group, mean (SD)		Between-groups effect			
	Intervention group (n=16)	Control group (n=39)	Cohen’s *d* (95% CI)	ANCOVA^b^		
				*t*_53_	*P*	Partial *η* ^*2*^
PDSS-SR	4.6 (3.3)	7.5 (4.2)	0.73 (–0.60, 2.32)	–2.60	.01	.115
BAI	15.6 (13.4)	22.6 (11.2)	0.60 (–2.93, 7.15)	–2.37	.02	.098
CES-D	12.1 (8.5)	21.6 (11.0)	0.94 (–2.50, 5.10)	–2.52	.02	.109

^a^Control group completers are those who provided posttreatment data. Intervention group completers are those who provided posttreatment data and completed at least lesson 4.

^b^Controlling for symptom severity at T0.

## Discussion

### Overview

This study showed that the Internet-based, guided, self-help intervention Don’t Panic Online was not effective in individuals with panic symptoms. Completers-only analyses did show moderate to large effect sizes between groups in favor of the intervention group. Adherence to the treatment was low. An analysis of the data using a less conservative imputation method revealed significant effects between groups in terms of the scores for general anxiety and depressive symptoms, but not for panic symptoms. Overall, the results show that Don’t Panic Online could be efficacious for intervention completers, but that it is not generally effective.

### Comparison With the Literature

A meta-analysis revealed that the psychological treatment (offline and online) of full-blown PD is highly effective compared to a waiting-list control group, with a mean effect size of *d*=1.19 [8]. Samples in which more than 50% of the participants had comorbid disorders did not benefit as much, but they still showed a large effect size even when compared with pooled active and nonactive control groups (*d*=0.83) [8]. Self-help interventions have an average effect size of *d*=0.75, again when compared with pooled control groups [8]. The results of our completers-only analyses are in-line with these findings. Treatment adherence is not reported in this meta-analysis, only study dropout rates, which averaged 9.53% for intervention groups.

For study design and intervention, our study is comparable with the trial of Meulenbeek et al [10]. That study found a moderate effect size of *d*=0.68 for the face-to-face group course Don’t Panic, an intervention with similar content to Don’t Panic Online. Treatment completion, defined as having followed at least 6 of the 8 sessions, was 75%. In that study, the participants had a relatively low baseline mean PDSS-SR score (7.2), which is similar to our study’s findings. Aside from panic symptoms, however, the sample differed from ours in a number of ways. Meulenbeek et al excluded participants with severe disorders other than PD, as well as those with social problems, and those who were receiving treatment for panic symptoms. In general, group interventions are no more effective than guided self-help interventions [38]. Possibly, any differences in outcome between the trials of Don’t Panic and Don’t Panic Online might be attributed to inclusion criteria.

Previous studies that compared Internet-based guided self-help for panic symptoms with a control group showed an overall effect size of Hedge’s *g*=0.83 [12]. Similar to Don’t Panic Online, the interventions studied were based on cognitive behavioral therapy and were similar in length [14-16]. Compared with these studies, effect sizes in the current study were expected a priori to be lower. We included a less severe group, thereby ruling out large decreases in symptom severity. Accordingly, assuming that there was no deterioration in the control group, the difference between the intervention group and control group at T1 could not be as large. With regard to low treatment adherence, this was not found in previous studies and values ranged from 79% to 95% [12,15].

There are several differences between our study and previous studies that may have had an impact on adherence. Firstly, all participants in our trial were free to use medication and find other treatment. Some may have found other help and decided to quit Don’t Panic Online. Secondly, our participants reported difficulties accessing the website. Thirdly, previous researchers had more telephone contact with their participants [14,15]. Our participants were also not interviewed after the treatment, whereas a scheduled interview after treatment may have led to better adherence [39]. Fourthly, the intervention we studied was not the same as the interventions of other studies. Perhaps Don’t Panic Online is not as effective or attractive as those examined in other studies. Lastly, our sample included a large proportion of participants with comorbid disorders, and possibly a proportion of participants who did not have PD as primary diagnosis. Perhaps an Internet-based intervention specifically for panic symptoms is less suited to this group. However, epidemiological data show that panic symptoms often coincide with psychiatric disorders other than PD [2,4]. Therefore, the participants of our study appear to be a representative sample of individuals with panic symptoms.

In summary, both clinical effect and treatment adherence were lower in our study than in previous studies of Internet-based self-help interventions and the Don’t Panic group course. The differences in sample characteristics between our study and previous trials could indicate that Internet-based interventions for panic symptoms are efficacious, but they may not be effective for all individuals seeking help for panic symptoms.

### Limitations

When interpreting our results, several limitations should be taken into account. One limitation of this study is nonresponse at the posttreatment measurement. For a large proportion of participants, it is unknown whether their panic symptoms increased, decreased, or remained stable. These missing values were estimated by multiple imputation. Although this can be considered a conservative imputation method, it is unlikely that the imputed values greatly underestimate the intervention effect. This is because many of the participants who did not respond at T1 also left the intervention after 1 or 2 sessions, and are, therefore, unlikely to have gained much benefit from it. A second limitation is that the intervention completers are small in number and may not be representative of the intervention group as a whole, even though there did not appear to be significant differences between completers and noncompleters. The comparison of this select group with the control group, for completers-only analyses, should be interpreted with caution. Thirdly, the control group could have had gained some benefit from the information website, which could have decreased the difference between T1 mean scores of the intervention group and control group. If that is the case, our study proved that Don’t Panic Online has, in general, no added value compared with an information website and our conclusion would remain the same. A fourth limitation is the lack of a follow-up measurement. It is not known whether the participants in either the intervention group or the control group showed any further improvement over the subsequent months to a year. Finally, all continuous measures were obtained by online self-report. The PDSS-SR could potentially yield lower mean scores than the PDSS interview [26], whereas online versions of questionnaires could potentially yield higher mean scores than pencil-and-paper versions [40,41]. These differences in psychometric properties limit the comparison of this study with other studies. However, this imposes no restrictions on comparisons between the intervention group and control group within our own study and, additionally, online and pencil-and-paper versions of panic questionnaires do appear to be equivalent [42,43].

### Implications and Future Research

Although previous research indicates that Internet interventions can be an efficacious treatment of panic symptoms, our results may suggest that a linear program targeting specific symptoms is not always effective. As our study and others have shown, panic symptoms generally coincide with comorbid symptoms. Therefore, transdiagnostic and tailorable interventions could be a future direction of Internet-based treatment of panic. Internet-based transdiagnostic self-help programs, tailored to the anxiety and/or depressive symptoms of the participant, show promising results in terms of the treatment of panic and other common mental disorders [13,44,45]. Tailored interventions could be more effective for individuals with higher symptom severity and comorbidity rates than nontailored programs [46]. Tailoring might help to increase treatment adherence because participants would then only see those sections that are applicable to them. Given the results of our study, the further development of transdiagnostic and tailorable Internet interventions should be encouraged.

Future research could focus on identifying those groups for whom Internet-based self-help interventions are effective, for example, by means of predictor and mediator analyses. Further research is also needed to investigate ways of boosting treatment adherence to Don’t Panic Online, of making it a feasible intervention for mild to moderate panic symptoms, and perhaps of modifying it to become more tailored and transdiagnostic in nature. This was the first study of Internet-based guided self-help for mild panic symptoms and our study needs to be replicated before we can draw any definitive conclusions. Lastly, although the efficacy of Internet-based guided self-help interventions has been established in several studies, it should be encouraged to conduct more pragmatic RCTs to examine the effectiveness.

## Multimedia Appendix 1

CONSORT-EHEALTH checklist V1.6.2 [18].

## Abbreviations

ANCOVA: analysis of covariance
BAI: Beck Anxiety Inventory
CIDI: Composite International Diagnostic Interview
EM: Expectation Maximization
ITT: intention-to-treat
MDD: major depressive disorder
MINI: Mini-International Neuropsychiatric Interview
PD: panic disorder
PDSS-SR: Panic Disorder Severity Scale-Self Report
RCT: randomized controlled trial
T0: baseline
T1: posttreatment assessment (12 weeks after T0)
