# Supplementary material for: An Internet-Based Guided Self-Help Intervention for Panic Symptoms: Randomized Controlled Trial
Source: J Med Internet Res. 2013 Jul 29;15(7):e154. doi: 10.2196/jmir.2362 (PMC3742403; doi:10.2196/jmir.2362)
Supplement: Supplementary file 1 [file jmir_v15i7e154_app1.pdf]

CONSORT-EHEALTH Checklist V1.6.2 Report

(based on CONSORT-EHEALTH V1.6), available at [http://tinyurl.com/consort-ehealth-v1-6].

2362

Date completed

11/30/2012 15:32:21

by

Wouter van Ballegooijen

Low-intensity treatment for panic symptoms – A pragmatic randomised controlled trial of an internet-based guided self-help intervention.

TITLE

1a-i) Identify the mode of delivery in the title

"an internet-based guided self-help intervention."

1a-ii) Non-web-based components or important co-interventions in title

Our intervention contained no non-web-based components.

1a-iii) Primary condition or target group in the title

"Low-intensity treatment for panic symptoms"

ABSTRACT

1b-i) Key features/functionalities/components of the intervention and comparator in the METHODS section of the ABSTRACT

"To evaluate the effectiveness of Don't Panic Online, an internet-based self-help course for mild panic symptoms, which is based on cognitive behavioural principles and includes guidance by email. (...) Participants (n = 126) were recruited from the general population and randomised to either the intervention group or to a waiting-list control group."

1b-ii) Level of human involvement in the METHODS section of the ABSTRACT

"Don't Panic Online, an internet-based self-help course for mild panic symptoms, which (...) includes guidance by email. (...) At baseline, diagnoses were obtained by interviews by telephone."

1b-iii) Open vs. closed, web-based (self-assessment) vs. face-to-face assessments in the METHODS section of the ABSTRACT

"Participants (n = 126) were recruited from the general population and randomised to either the intervention group or to a waiting-list control group."

"Measurements were conducted online and took place at baseline and 12 weeks after baseline (T1). At baseline, diagnoses were obtained by interviews by telephone."

1b-iv) RESULTS section in abstract must contain use data

"Analyses of covariance (intention-to-treat) showed no significant differences in panic symptom reduction between groups. Completers-only analyses revealed a moderate effect size in favour of the intervention group (d = 0.73, P = .012). Only 27% of the intervention group finished lesson 4 or more (out of 6). Non-response at T1 was high for the total sample (42%)."

1b-v) CONCLUSIONS/DISCUSSION in abstract for negative trials

We report the high level of non-completion: "Only 27% of the intervention group finished lesson 4 or more (out of 6)."

However, we do not state any potential reasons for this high level of non-completion in the abstract. There are many possible reasons and it would be too speculative to pick just a few factors.

INTRODUCTION

2a-i) Problem and the type of system/solution

"Panic disorder (PD) with or without agoraphobia is a prevalent anxiety disorder associated with substantial loss of quality of life for the patient and considerable costs to society [1-4]. Just as prevalent is subclinical PD [2, 4], which can be defined as panic symptoms that do not meet full DSM-IV criteria for PD. Subclinical panic symptoms can develop into clinical PD and are also a predictor for the development of mental disorders other than PD, such as generalised anxiety disorder, social phobia or major depressive disorder (MDD) [5]."

"Internet-based guided self-help has shown to be an efficacious treatment of PD as well, with a large effect size (Hedge's g = 0.83) [12]. To date, all but one study [13] comparing internet-based guided self-help for PD with a control condition have focussed purely on groups with clinical PD, which, commonly, also had to be the primary diagnosis, e.g. [14-15]. These studies excluded subclinical cases, e.g. [14-16]. Recently, an internet-based version of the group course Don't Panic has been developed. This intervention, Don't Panic Online (DPO), is an internet-based self-help course with minimal guidance, specifically for individuals with mild panic symptom severity. The aim was to provide an accessible, low-intensity, early intervention for panic symptoms."

2a-ii) Scientific background, rationale: What is known about the (type of) system

See previous item about the rationale.

Potential impact of findings: to find out whether internet-based self-help could be an effective treatment for mild cases of panic.

The comparator is a waiting-list control group with access to an information website. This type of control group meets both ethical and methodological standards.

METHODS

3a) CONSORT: Description of trial design (such as parallel, factorial) including allocation ratio

"The current study is a pragmatic randomised controlled trial (RCT) of the effectiveness of DPO in reducing panic and anxiety symptoms among participants with subclinical and mild clinical PD. We postulate a difference in effect between DPO and a waiting-list control group."

3b) CONSORT: Important changes to methods after trial commencement (such as eligibility criteria), with reasons

Not applicable.

3b-i) Bug fixes, Downtimes, Content Changes

No, not in the methods section. There have been technical difficulties, but this has been reported in the Results section.

4a) CONSORT: Eligibility criteria for participants

We included participants aged 18 and above, with subclinical PD or clinical PD with relatively mild symptom severity, and access to the internet. Any individuals who were at risk of suicide were excluded. Subclinical or mild PD was defined as having a score of 5 to 15 on the Panic Disorder Severity Scale-Self Report (PDSS-SR) [19]. These cut-off points represent slight to moderate panic symptom severity [20]. No restrictions were imposed on the use of pharmacotherapy or psychotherapy.

4a-i) Computer / Internet literacy

Yes. Participants were required to have "access to the internet".

4a-ii) Open vs. closed, web-based vs. face-to-face assessments:

"Participants were recruited from the general population. Most of those who applied for participation did so after reading about this study in the health section of an online newspaper. Additional online recruitment was conducted by means of a Facebook advertising campaign and by posting messages on panic-related or anxiety-related message boards. This was supplemented by 'offline' recruitment by means of advertisements in national newspapers and articles in local newspapers. Interested individuals were directed to a study website, where they could find information about participation"

"applicants were sent an email with a link to the online questionnaires. The baseline (T0) questionnaires included the screening questionnaires for inclusion. (...) Those participants who had completed T0 and who met the inclusion criteria were contacted within two weeks for a diagnostic interview by telephone. This interview was used to obtain a more detailed overview of the study sample, not for the purposes of inclusion or exclusion."

4a-iii) Information giving during recruitment

"Interested individuals were directed to a study website, where they could find information about participation, and a downloadable informed consent form."

The study website explained the study design in layman terms.

4b) CONSORT: Settings and locations where the data were collected

"Those participants who had completed T0 and who met the inclusion criteria were contacted within two weeks for a diagnostic interview by telephone. (...) Both T0 and T1 were self-report and were conducted through the internet."

The researchers conducted the study at VU University Amsterdam and the Netherlands instititute of mental health and addiction (Trimbos-institute), Utrecht, The Netherlands.

4b-i) Report if outcomes were (self-)assessed through online questionnaires

Yes, see the previous item.

4b-ii) Report how institutional affiliations are displayed

5) CONSORT: Describe the interventions for each group with sufficient details to allow replication, including how and when they were actually administered

5-i) Mention names, credential, affiliations of the developers, sponsors, and owners

The intervention was developed by the Trimbos-institute, which is the Netherlands Institute for Mental health and Addiction, in collaboration with GGNet, a Dutch mental health care institute.

5-ii) Describe the history/development process

5-iii) Revisions and updating

5-iv) Quality assurance methods

5-v) Ensure replicability by publishing the source code, and/or providing screenshots/screen-capture video, and/or providing flowcharts of the algorithms used

5-vi) Digital preservation

5-vii) Access

The participants could access the intervention at home or at any other place where they had access to the internet. They were not paid for their participation, nor were they required to pay.

5-viii) Mode of delivery, features/functionalities/components of the intervention and comparator, and the theoretical framework

The course consists of six sessions, in which the participants learn to control their panic symptoms by applying various cognitive behavioural techniques and skills. The intervention consists of the following components: keeping a log of panic attacks; analysis of fearful situations; challenging thoughts that enable feelings of panic; replacing these thoughts by more realistic, constructive thoughts that reduce anxiety; behavioural exercises; and ranking exercises from manageable to difficult and carrying them out in order of difficulty. Each session consists of text, voice over, animated diagrams and video. A typical session will take about thirty minutes and consists of an introduction, a discussion of the previous lesson’s homework, new theory and homework for the next week. The course is designed to be followed on a weekly basis until session five, while the sixth lesson can be followed four weeks after the fifth. The course can be completed in eight weeks.

Besides the lessons, the participants has several online resources at his or her disposal: a homework station, a panic attack log, a library for extra information, reading tips and a discussion board. A track-and-trace system keeps a record of the dates on which participants log on and complete a lesson.

Coaching was conducted by email and was, therefore, asynchronous.

5-ix) Describe use parameters

5-x) Clarify the level of human involvement

5-xi) Report any prompts/reminders used

"Every week, [participants in the intervention group] received an email from their coach"

Concerning reminders for the trial: "Any participants who had not completed T0 or T1 were sent up to three automated reminders by email, at weekly intervals."

5-xii) Describe any co-interventions (incl. training/support)

There were no co-interventions.

6a) CONSORT: Completely defined pre-specified primary and secondary outcome measures, including how and when they were assessed

Primary outcome measures:
Panic symptom severity, measured using the panic disorder severity scale - self-report (PDSS-SR).
Anxiety symptom severity, measured using the Beck Anxiety Inventory (BAI).

Secondary outcome measures:
Depressive symptom severity, measured using the CES-D
Treatment completion and engagement

6a-i) Online questionnaires: describe if they were validated for online use and apply CHERRIES items to describe how the questionnaires were designed/deployed
Of our outcome measures, only the CES-D has been validated for online use.
6a-ii) Describe whether and how "use" (including intensity of use/dosage) was defined/measured/monitored
A track-and-trace system kept a record of the dates on which participants logged on and completed a lesson.
6a-iii) Describe whether, how, and when qualitative feedback from participants was obtained
"the T1 battery of online questionnaires included open questions concerning the participant's subjective experience with DPO and reasons for not finishing the programme."
6b) CONSORT: Any changes to trial outcomes after the trial commenced, with reasons
We also administered self-report measures for quality of life, production loss and health care usage. Except for health care usage at baseline, these measure are not reported in the main paper of this trial. We had planned a cost-effectiveness evaluation, but dropped that idea for the main paper.
7a) CONSORT: How sample size was determined
7a-i) Describe whether and how expected attrition was taken into account when calculating the sample size
"Previous RCTs of internet-based self-help interventions for panic symptoms showed large between-group effect sizes [12]. Our aim was to recruit participants with milder symptom severity than those who took part in these studies. Therefore, our sample was expected to show a smaller decrease in panic symptoms. Based on a moderate effect size (Cohen's d = 0.50), and using a two-sided t-test ($\alpha = .05$, power 80%) to compare the PDSS-SR scores of the intervention group with those of the control group, we aimed to include 128 participants [21], with 64 in each group. Any missing values at post-treatment were imputed."
7b) CONSORT: When applicable, explanation of any interim analyses and stopping guidelines
Not applicable.
8a) CONSORT: Method used to generate the random allocation sequence
"Randomisation lists were generated automatically, using a computer program."
8b) CONSORT: Type of randomisation; details of any restriction (such as blocking and block size)
"Randomisation was stratified for the presence or absence of agoraphobic symptoms (PDSS-SR item 4 score 2 or higher) and the use of antidepressants or sedatives."
9) CONSORT: Mechanism used to implement the random allocation sequence (such as sequentially numbered containers), describing any steps taken to conceal the sequence until interventions were assigned
"Randomisation lists were generated automatically, using a computer program."
10) CONSORT: Who generated the random allocation sequence, who enrolled participants, and who assigned participants to interventions
"Randomisation lists were generated automatically, using a computer program."

When a participant was assigned to one of the two groups, one of the researchers sent him/her an email with the randomisation result and access codes.
11a) CONSORT: Blinding - If done, who was blinded after assignment to interventions (for example, participants, care providers, those assessing outcomes) and how
11a-i) Specify who was blinded, and who wasn't
"The T0 measurement can be considered to be double blind, as the participants were not randomised until they had completed all of the questionnaires and the diagnostic interview. Blinding of the participants at post-treatment assessment (T1) was not possible, as at that stage they were aware of the nature of the group to which they had been allocated."
11a-ii) Discuss e.g., whether participants knew which intervention was the "intervention of interest" and which one was the "comparator"
This is not litterally reported in our paper. However, the participants were aware of the intervention of interest, because they had read the information about the study design before they had signed their infomred consent form.
11b) CONSORT: If relevant, description of the similarity of interventions
not applicable
12a) CONSORT: Statistical methods used to compare groups for primary and secondary outcomes
"Between-group effects at T1 were calculated using analyses of covariance (ANCOVA), controlling for pre-treatment scores. Effect sizes on continuous measures were expressed in terms of Cohen's d, which was calculated by subtracting one mean score from the other, and dividing the mean difference thus obtained by the pooled standard deviation."

"Within-group effects were analysed using paired-sample t-tests, and expressed in terms of Cohen's d, where the correlation between T0 and T1 was taken into account by applying Morris & DeShon's equation 8 [34]. Finally, the proportion of participants below the PDSS-SR cut-off points for clinical and subclinical PD was calculated for both T0 and T1. We used the cut-off points of 8 and 5, the former indicating clinical PD [24] and the latter subclinical PD [20]. (...) We maintained a two-sided α of .05. SPSS version 17 was used for all analyses."
12a-i) Imputation techniques to deal with attrition / missing values
"The data were analysed in agreement with the intention to treat (ITT) principle. Missing data at T1 were imputed by multiple imputation, where, except for nominal variables, all variables (i.e. age, education level, clinical diagnoses and symptom severity on all measures at T0 and T1) were included as predictors. Ten datasets were generated and analyses were performed using pooled data. Compared with single imputation methods, multiple imputation generates a more conservative estimate of the sample standard error [35] and overestimation of effect sizes and P-values is unlikely. For the purpose of sensitivity analysis, P-values and effect sizes were also estimated by running the Expectation Maximisation (EM) algorithm [36] on the missing data."
12b) CONSORT: Methods for additional analyses, such as subgroup analyses and adjusted analyses
"All analyses were conducted for the full sample, for the subgroup completers, and for subgroups with and without the diagnosis of PD according to the CIDI."

RESULTS

13a) CONSORT: For each group, the numbers of participants who were randomly assigned, received intended treatment, and were analysed for the primary outcome
"Of 368 applicants who applied and sent in informed consent forms, 126 were included in the study."

Further information is given in the tables of our paper.
13b) CONSORT: For each group, losses and exclusions after randomisation, together with reasons
This is shown in our flow chart.
13b-i) Attrition diagram
Yes, it is shown in our flow chart.
14a) CONSORT: Dates defining the periods of recruitment and follow-up

The time between baseline and post-treatment measurements has been reported:

"T1 was scheduled twelve weeks after the baseline assessment."

The exact dates have not been reported in our paper. The recruitment of participants took place from March 2010 to December 2010.

14a-i) Indicate if critical “secular events” fell into the study period

14b) CONSORT: Why the trial ended or was stopped (early)

The study has been completed according to plan. Further follow-up measurements could have been interesting, but were not possible due to a lack of time and funds.

15) CONSORT: A table showing baseline demographic and clinical characteristics for each group

See Table 1.

15-i) Report demographics associated with digital divide issues

Age, education, gender and employment status have been reported. Internet literacy has not been reported.

16a) CONSORT: For each group, number of participants (denominator) included in each analysis and whether the analysis was by original assigned groups

16-i) Report multiple “denominators” and provide definitions

This is quite clear in the Tables 2 and 3.

16-ii) Primary analysis should be intent-to-treat

"Intention to treat analyses
After multiple imputation, analyses of covariance showed no significant difference in panic symptom severity at T1 between groups as measured by the PDSS-SR ($t = -1.17$, $P = .246$, partial $\eta 2 = .023$, Cohen's $d = 0.30$; Table 2). The within-group difference of the intervention group was significant ($t = 3.06$, $P = .007$, $d = 0.62$), as was the within-group difference of the control group, albeit with a smaller effect size ($t = 2.26$, $P = .033$, $d = 0.40$). The mean BAI score, too, did not differ between groups ($t = -1.71$, $P = .087$, partial $\eta 2 = .027$, $d = 0.39$; Table 2)."

17a) CONSORT: For each primary and secondary outcome, results for each group, and the estimated effect size and its precision (such as 95% confidence interval)

"After multiple imputation, analyses of covariance showed no significant difference in panic symptom severity at T1 between groups as measured by the PDSS-SR ($t = -1.17$, $P = .246$, partial $\eta 2 = .023$, Cohen's $d = 0.30$; Table 2). The within-group difference of the intervention group was significant ($t = 3.06$, $P = .007$, $d = 0.62$), as was the within-group difference of the control group, albeit with a smaller effect size ($t = 2.26$, $P = .033$, $d = 0.40$). The mean BAI score, too, did not differ between groups ($t = -1.71$, $P = .087$, partial $\eta 2 = .027$, $d = 0.39$; Table 2). Nor were there any differences between groups in terms of depressive symptoms, as measured by the CES-D ($t = -1.56$, $P = .123$, partial $\eta 2 = .034$, $d = 0.39$; Table 2).
At T1, and with missing values imputed, 24 participants (38%) in the intervention group and 13 (20%) in the control group had PDSS-SR scores of less than 5, i.e. symptom free. This difference did not reach significance ($X2 = 5.70$, $P = .117$). With regard to the cut-off point of 8 (the recommended cut-off for clinical diagnosis), 28 participants (44%) in the intervention group and 22 (35%) in the control group scored below 8 at T0. At T1, 38 participants in the intervention group (60%) and 33 participants in the control group (52%) scored below 8, a non-significant difference ($X2 = 1.34$, $P = .53$)."

17a-i) Presentation of process outcomes such as metrics of use and intensity of use

17b) CONSORT: For binary outcomes, presentation of both absolute and relative effect sizes is recommended

There are only few binary outcomes. In the intention-to-treat analyses, none of these were significant. The effect sizes were not reported.

18) CONSORT: Results of any other analyses performed, including subgroup analyses and adjusted analyses, distinguishing pre-specified from exploratory

Participants with a diagnosis of PD versus those with no diagnosis:
"Neither ITT nor completers-only analyses showed differences, on any outcome measure, between participants with and without clinical PD."

18-i) Subgroup analysis of comparing only users

Completers-only analyses:
"Those participants in the intervention group who had completed the first four lessons (or more) of the course (n = 17) were included in the completers-only analyses. These 'completers' cannot be all considered to have completed the intervention, but after 4 lessons, participants can be considered to have experienced most of the content of the intervention. Sixteen of the 17 participants in the intervention group who had completed the first four lessons also filled in T1 questionnaires. Accordingly, there were 16 completers in the intervention group. These 16 individuals did not significantly differ from the non-completers in the intervention group at T0 in terms of age, education, clinical diagnosis and symptom severity. Control group 'completers' were those who filled in T1 (n = 39).
Analyses of covariance showed significant differences between the intervention group completers and control group completers with regard to panic symptom severity at T1 ($t = -2.60$, $P = .012$, $d = 0.73$; see Table 3), in favour of the intervention group. The intervention group was also characterised by a large within-group effect on panic symptoms ($t = 4.92$, $P < .001$, $d = 1.23$). In the control group, within-group effects did not reach significance.
Analyses of covariance also showed that BAI anxiety symptom severity differed significantly between groups ($t = -2.37$, $P = .021$, $d = 0.60$, see Table 3), as did depressive symptom severity, as measured using the CES-D ($t = -2.52$, $P = .015$, $d = .94$)."

The limitations of the completers-only analyses are addressed in the Discussion section of our paper.

19) CONSORT: All important harms or unintended effects in each group

Not applicable.

19-i) Include privacy breaches, technical problems

Technical propblems:
"During the trial, several participants reported that they experienced difficulties accessing the website."

19-ii) Include qualitative feedback from participants or observations from staff/researchers

"Those participants in the intervention group who completed T1 but did not complete the intervention (n = 30) were asked why they dropped out. The most frequently reported reasons involved time constraints (n = 13), life events (n = 5), and symptoms so severe that the individual was unable to follow the programme (or parts thereof) or carry out the assignments (n = 5; see Table 5)."

DISCUSSION

20) CONSORT: Trial limitations, addressing sources of potential bias, imprecision, multiplicity of analyses

20-i) Typical limitations in ehealth trials

"One limitation of this study is non-response at the post-treatment measurement. For a large proportion of participants, it is unknown whether their panic symptoms increased, decreased, or remained stable. These missing values were estimated by multiple imputation. While this can be considered a conservative imputation method, it is unlikely that the imputed values greatly underestimate the intervention effect. This is because many of the participants who did not respond to T1 also left the intervention after one or two sessions, and are therefore unlikely to have gained much benefit from it. A second limitation is that the intervention completers are small in number and may not be representative of the intervention group as a whole, even though there did not appear to be significant differences between completers and non-completers. The comparison of this select group with the control group, for completers-only analyses, should be interpreted with caution. Thirdly, the control group could have had gained some benefit from the information website, which could have decreased the difference between T1 mean scores of the intervention group and control group. If that is the case, our study proved that DPO has, in general, no added value compared with an information website and our conclusion would remain the same. A fourth limitation is the lack of a follow-up measurement. It is not known whether the participants in either the intervention group or the control group showed any further improvement over the subsequent months to a year. Finally, all continuous measures were obtained by online self-report. The PDSS-SR could potentially yield lower mean scores than the PDSS interview [25], while online versions of questionnaires could potentially yield higher mean scores than pencil-and-paper versions [39-40]. These differences in psychometric properties limit the comparison of this study with other studies. However, this imposes no restrictions on comparisons between the intervention group and control group within our own study and, additionally, online and pencil-and-paper versions of panic questionnaires do appear to be equivalent [41-42]."

21) CONSORT: Generalisability (external validity, applicability) of the trial findings

21-i) Generalizability to other populations

"our sample included a large proportion of participants with comorbid disorders, and possibly a proportion of participants who did not have PD as primary diagnosis. Perhaps an internet-based intervention specifically for panic symptoms is less suited to this group. However, epidemiological data show that panic symptoms often coincide with psychiatric disorders other than PD [2, 4]. Therefore, the participants of our study appear to be a representative sample of individuals with panic symptoms."

"This was the first study of internet-based guided self-help for mild panic symptoms and our study needs to be replicated before we can draw any definitive conclusions."

21-ii) Discuss if there were elements in the RCT that would be different in a routine application setting

22) CONSORT: Interpretation consistent with results, balancing benefits and harms, and considering other relevant evidence

22-i) Restate study questions and summarize the answers suggested by the data, starting with primary outcomes and process outcomes (use)

"This study showed that the internet-based guided self-help intervention Don't Panic Online (DPO) was not effective in individuals with panic symptoms. Completers-only analyses did show moderate to large effect sizes between groups, in favour of the intervention group. Adherence to the treatment was low. An analysis of the data using a less conservative imputation method revealed significant effects between groups in terms of the scores for general anxiety and depressive symptoms, but not for panic symptoms. Overall, the results show that DPO could be efficacious for intervention completers, but that it is not generally effective."

22-ii) Highlight unanswered new questions, suggest future research

"Future research could focus on identifying those groups for whom internet-based self-help interventions are effective, by means of predictor and mediator analyses, for example. Further research is also needed to investigate ways of boosting treatment adherence to DPO, of making it a feasible intervention for mild to moderate panic symptoms, and perhaps of modifying it to become more tailored and transdiagnostic in nature."

Other information

23) CONSORT: Registration number and name of trial registry

This trial has been registered in the Netherlands Trial Register (NTR1639). The Netherlands Trial Register is part of the Dutch Cochrane Centre.

24) CONSORT: Where the full trial protocol can be accessed, if available

Van Ballegooijen W, Riper H, van Straten A, Kramer J, Conijn B, Cuijpers P. The effects of an Internet based self-help course for reducing panic symptoms--Don't Panic Online: study protocol for a randomised controlled trial. Trials. 2011;12:75.

25) CONSORT: Sources of funding and other support (such as supply of drugs), role of funders

"This study is funded by the Trimbos Institute and VU University Amsterdam."

X26-i) Comment on ethics committee approval

Approval by the local ethics committee has been reported. Ethical considerations have not been specifically reported, except how we dealt with applicants who expressed a suicide risk:

"Any participants who reported severe panic symptoms or who were at risk of suicide were sent an automatic message advising them to contact their general practitioner and/or to visit a website for suicide prevention. This website (www.113online.nl) offers psycho-education and a help-line by telephone or online chat"

x26-ii) Outline informed consent procedures

"Interested individuals were directed to a study website, where they could find information about participation, and a downloadable informed consent form. The application procedure involved printing and signing the informed consent form, then sending this to the research team (either as a physical document, by conventional mail, or as a scanned document attached to an email)."

X26-iii) Safety and security procedures

X27-i) State the relation of the study team towards the system being evaluated

"The authors declare no conflict of interest."
